# Tissue sensitivity to thyroid hormones may change over time

**DOI:** 10.1530/ETJ-21-0054

**Published:** 2022-01-21

**Authors:** Giorgio Radetti, Franco Rigon, Alessandro Salvatoni, Irene Campi, Tiziana De Filippis, Valentina Cirello, Silvia Longhi, Fabiana Guizzardi, Marco Bonomi, Luca Persani

**Affiliations:** 1 Marienklinik, Bolzano, Italy; 2 Department of Paediatrics, University of Padua, Padua, Italy; 3 Department of Medicine and Surgery, University of Insubria, Varese, Italy; 4 Division of Endocrine and Metabolic Diseases and Laboratory of Endocrine and Metabolic Research, Istituto Auxologico Italiano, IRCCS, Milan, Italy; 5 Department of Paediatrics, Regional Hospital of Bolzano, Bolzano, Italy; 6 Department of Medical Biotechnology and Translational Medicine, University of Milan, Milan, Italy

**Keywords:** resistance to thyroid hormones, hyperthyroidism, hypothyroidism, Refetoff syndrome

## Abstract

**Introduction:**

Patients with congenital hypothyroidism (CH) may transiently show a certain degree of pituitary resistance to levothyroxine (LT4) which, however, normalizes subsequently. However, in some individuals, thyroid-stimulating hormone (TSH) fails to normalize despite adequate LT4 treatment.

**Methods:**

Nine patients with CH followed in three Academic Centre who developed over time resistance to thyroid hormones underwent extensive biochemical and genetic analyses. These latter were performed by Sanger sequence or targeted next-generation sequencing technique including a panel of candidate genes involved in thyroid hormone actions and congenital hypothyroidism (CH): *THRA, THRB, DIO1, DIO2, SLC16A2, SECISBP2, DUOX2, DUOXA2, FOXE1, GLIS3, IYD, JAG1, NKX2-1, NKX2-* *5, PAX8, SLC26A4, SLC5A5, TG, TPO, TSHR*.

**Results:**

All patients displayed a normal sensitivity to thyroid hormone (TH) in the first years of life but developed variable degrees of resistance to LT4 treatment at later stages. In all cases, TSH normalized only in the presence of high free thyroxine levels. Tri-iodothyronine suppression test followed by thyrotrophin-releasing hormone stimulation was performed in two cases and was compatible with central resistance to THs. This biochemical feature was present independently on the cause of CH, being observed either in patients with an ectopic (*n* = 2) or eutopic gland (*n* = 3) or in case of athyreosis (*n* = 1). None of the patients had genetic variants in genes involved in the regulation of TH actions, while in two cases, we found two double heterozygous missense variants in *TSHR* and *GLIS3* or in *DUOX2* and *SLC26A4* genes, respectively.

**Conclusions:**

We report CH patients who showed an acquired and unexplainable pituitary refractoriness to TH action.

## Introduction

Patients with congenital hypothyroidism (CH) display a certain degree of resistance to levothyroxine (LT4) either at birth or during childhood (1, 2). This clinical picture usually improves later in life (1), although adult CH patients maintain euthyroidism with higher LT4 doses compared to adulthood-acquired hypothyroidism (3). The reasons for this condition are unknown, but a genetic origin appears unlikely, as it has been observed either in patients with ectopic or eutopic thyroid gland (1). The concomitant occurrence of CH and resistance to thyroid hormone syndrome type β (RTHβ) are exceptional, since only five patients have been described so far, and only in four patients, a mutation in the *THRB* gene has been demonstrated (4, 5, 6, 7).

RTHβ, firstly described by Refetoff in 1967 (8), is characterized by elevated circulating free thyroxine (fT4) and free tri-iodothyronine (fT3) together with an unsuppressed thyroid-stimulating hormone (TSH). RTHβ is caused by dominant negative genetic variants in the *THRB,* resulting in a mutant thyroid hormone receptor beta (TRβ) with an impaired transactivating ability (9) while deletions of *THRB* gene are exceptional (10, 11). In a minority of cases, referred to as non-TR RTH, no molecular defects are found (12).

The clinical picture is highly variable (13) and it has been hypothesized that such variability may depend on a variable level of expression of the mutant allele (14, 15) or to a mosaicism of the mutant T3 receptors (16). In addition, the clinical picture might slightly change over time, presumably secondary to age-related changes in T3 receptor function (17).

Recently, Lacámara* et al.* reported resistance to exogenous thyroxine (RETH) (18) in patients with different causes of hypothyroidism, including total thyroidectomy. Differently from RTHβ patients, RETH patients had a decreased T3/T4 ratio, suggesting that D2 functional deficiency underlies RETH but not RTHβ.

In patients with CH, the percentage of patients showing central resistance to thyroid hormone (TH) decreases with age, while an acquired resistance, to our knowledge, has never been described.

Here, we report nine patients who showed over the years significant changes in tissue sensitivity to THs.

Differently from RETH patients, who display hyperthyroid symptoms with increasing LT4 dose, our patients were euthyroid even in the presence of high FT4 levels with a normal TSH, similarly to what reported in most patients with conventional RTHβ.

## Materials and methods

### Auxology

The Italian standards for age and gender (19) were used for the auxological evaluation.

### Assays

TSH and fT4 were measured using Elecsys 2010 electrochemiluminescence immunoassay analyzer (Cobas®, Roche Diagnostics GmbH); the intra- and inter-assay CVs for TSH were 2.7 and 3.2%, with a sensitivity limit of 0.005 mU/L. For fT4, the intra- and inter-assay CVs were 1.8 and 2.6%, respectively, and the limit of sensitivity was 0.103 pmol/L. TH was assayed on the two-step immunoassay DELFIA platform (PerkinElmer) in two cases during the T3 suppression test.

The intra- and inter-assay CV for fT4 were 3.1 and 6.9%, respectiveley, with an analytical sensitivity < 2 pmol/L and for FT3, intra- and inter-assay CV were 7.7 and 11.3%, respectively, with a analytical sensitivity of 1.5 pmol/L.

### Genetic analyses

Genomic DNA was extracted from peripheral blood lymphocytes by the BACC2 Nucleon kit (GE Healthcare Life Sciences) for direct sequencing and used Gene Catcher gDNA 96 × 10 mL Automated Blood kit (Invitrogen, Life Technologies™) for next-generation sequencing (NGS).

In four patients (#2, #3, #5, #6), we studied the following candidate genes: *SERPINA7, THRB, GLIS3*and* TSHR* by Sanger direct sequencing, using BigDyeVR Terminator v.3.1 Cycle Sequencing Kit (Life Technologies) on a 3100 DNA Analyzer (Applied Biosystems).

In the remaining patients, genetic investigations were performed by NGS technique.

The gene panel was designed using Illumina Design Studio and included 20 candidate genes involved in TH actions and CH: *THRA, THRB, DIO1, DIO2, SLC16A2, SECISBP2, DUOX2, DUOXA2, FOXE1, GLIS3, IYD, JAG1, NKX2-1, NKX2-5, PAX8, SLC26A4, SLC5A5, TG, TPO, TSHR*. The 20 genes that were consistently represented in all sequence capture panels were assessed for the purposes of this study. Libraries were prepared using Illumina Nextera Rapid Capture Custom Enrichment kits according to the manufacturer’s protocols. All regions that were not sequenced were recovered with NexteraVR DNA Library Preparation kit (Illumina). For subsequent analyses, we included as ‘rare variants’ all known pathogenic or rare non-synonymous or splicing-site variants (minor allele frequency, MAF ≤ 0.01) and novel non-synonymous or splicing-site variants. The frequency and the functional annotation of the identified variants were checked in public databases (Ensembl, UCSC Genome browser, 1000 Genome project, ExAC Browser, NCBI, HGMD professional, GnomAD), considering the ethnic groups (Europeans). As previously reported (20, 21), we excluded common non-synonymous variants with MAF > 0.01, synonymous, intronic and 5’ or 3’ UTR variants. Each variant found was confirmed by Sanger direct sequencing using BigDyeVR Terminator v.3.1 Cycle Sequencing Kit (Life Technologies) on a 3100 DNA Analyzer (Applied Biosystems).

All the subjects and their parents (or legal guardian) have given their written consent to participate in the study. The study was approved by the institute’s committee (Istituto Auxologico Italiano) on human research. This study protocol was reviewed and approved by Istituto Auxologico Italiano and Ministry of Health, CASI, approval number 02C502_2005.

### Case reports

All patients of this study had CH, due to structural abnormalities of the thyroid in five cases (thyroid hypoplasia, thyroid ectopia and thyroid athyreosis in one, two and two patients, respectively), while the remaining four patients had a functional defect with thyroid gland *in situ*. They were diagnosed at birth by the neonatal screening, which was based on TSH evaluation in all centres and promptly started on LT4 substitution at the commonly recommended doses. They were then followed according to clinical practice guidelines for CH, and on LT4 replacement treatment, FT4 and TSH levels normalized. Variants in genes associated with CH were detected in five out of nine patients (Table 1), and an oligogenic involvement was found in four of them.

**Table 1:** Clinical, biochemical and genetic data of the patients included in the study in the first years of life.

ID/sex	TSH at screening (mU/L)	Thyroid scan/US	Molecular analysis	Assessments performed in the first years of life			
				LT4 dose^a^ (µg/kg)	Expected dose (µg/kg)	TSH (mU/L)	fT4 (pmol/L)
#1/F	>100	Athyreosis	GLIS3 p.S143Y +* TSHR* p.T607I	8	10–15	3.3	18.9
#2/F	618	Normal	WT	9	8–10	3.2	20.2
#3/F	71	Normal	*DUOX2* p.E1546G + *SLC26A4* p.L597S	7	8–10	2.1	17.5
#4/M	361	Ectopia	WT	10	10–15	2.7	21.5
#5/F	>100	Normal	*TSHR* p.I155L	10	8–10	3.8	17.8
#6/F	270	Ectopia	WT	15	10–15	3.7	16.9
#7/F	11.9	Normal	*DUOX2 p*.G200R +* TPO* p.P906L	2.5	2–4	2.5	21.6
#8/M	557	Hypoplasia	WT	15	10–15	2.7	17.9
#9/F	384	Athyreosis	DUOX2 p.K1474E p.R1510H TG p.E1136Q p.R1136Q	12	10–15	4.2	18.1

^a^Dose required to maintain TSH within the normal range and fT4 in the upper half of the normal range during follow-up

LT4, levothyroxine.

Surprisingly, after a variable period of time (range, 1–12 years), an increased LT4 dosage was required to maintain TSH in the normal level, and eventually, even with fT4 level in the high normal range or above the upper limit, TSH remained inappropriately elevated (Table 2). The possibility of an incorrect intake or malabsorption of T4 was discarded because of the high serum fT4 levels, by the fact that the treatment has been always administered by the parents or under parental supervision since the altered profile was detected, by exclusion of coeliac disease (clinically and absence of endomysial and transglutaminase antibodies) and other causes of malabsorption. Analytical interferences with the immunoassays by macro-TSH, biotin, antistreptavidin antibodies, antiruthenium (−Ru) antibodies, TH autoantibodies (THAAbs) and heterophilic antibodies (22) were also excluded using different assays (23). None of the patients recovered from this condition even after a long follow-up (1.3–17.10 years after the onset of the resistance) and attempts to lower the doses towards the physiological ones. For all patients, the decision to maintain the fT4 serum levels in the high normal range was chosen, as the patients were clinically euthyroid and no signs of thyrotoxicosis were noted. Linear growth was regular and consistent with their expected genetic target, and they did not display any behavioural abnormality, apart from a mild cognitive impairment in one patient. Table 1 summarizes all the clinical data of the patients included in the study.

**Table 2:** Clinical and biochemical of the patients included in the study when RETH was discovered.

ID/sex	Assessments performed when RTH was discovered						Lowest TSH observed during the follow-up with the respective fT4 (TSH/fT4)
	Age at diagnosis of RETH (years)	TSH (mU/L)	fT4 (pmol/L)	fT3 (pmol/L)	LT4 dose (µg/kg)	fT4/fT3 ratio	
#1/F	12	16.4	19.5	3.8	2.3	5.1	12.9/21.9
#2/F	2	8.5	23.8	6.1	5	3.9	6.9/20.5
#3/F	3	7.9	21.2	6.9	8	3.1	8.5/19.4
#4/M	2	12	21.9	6.5	8	3.3	25.3/25.6
#5/F	1.2	46	16.8	5.9	7.5	2.9	31.7/20.1
#6/F	4	43	17.6	4.9	2.8	3.6	10/21.5
#7/F	3.5	7.22	20.9	5.1	2	4.1	7.5/22.2
#8/M	2	12	21.8	6.3	2.5	3.5	8.9/22.3
#9/F	1.3	22	16.9	4.8	2.6	3.5	14.7/20.6

### Case 1

**Figure 1:**
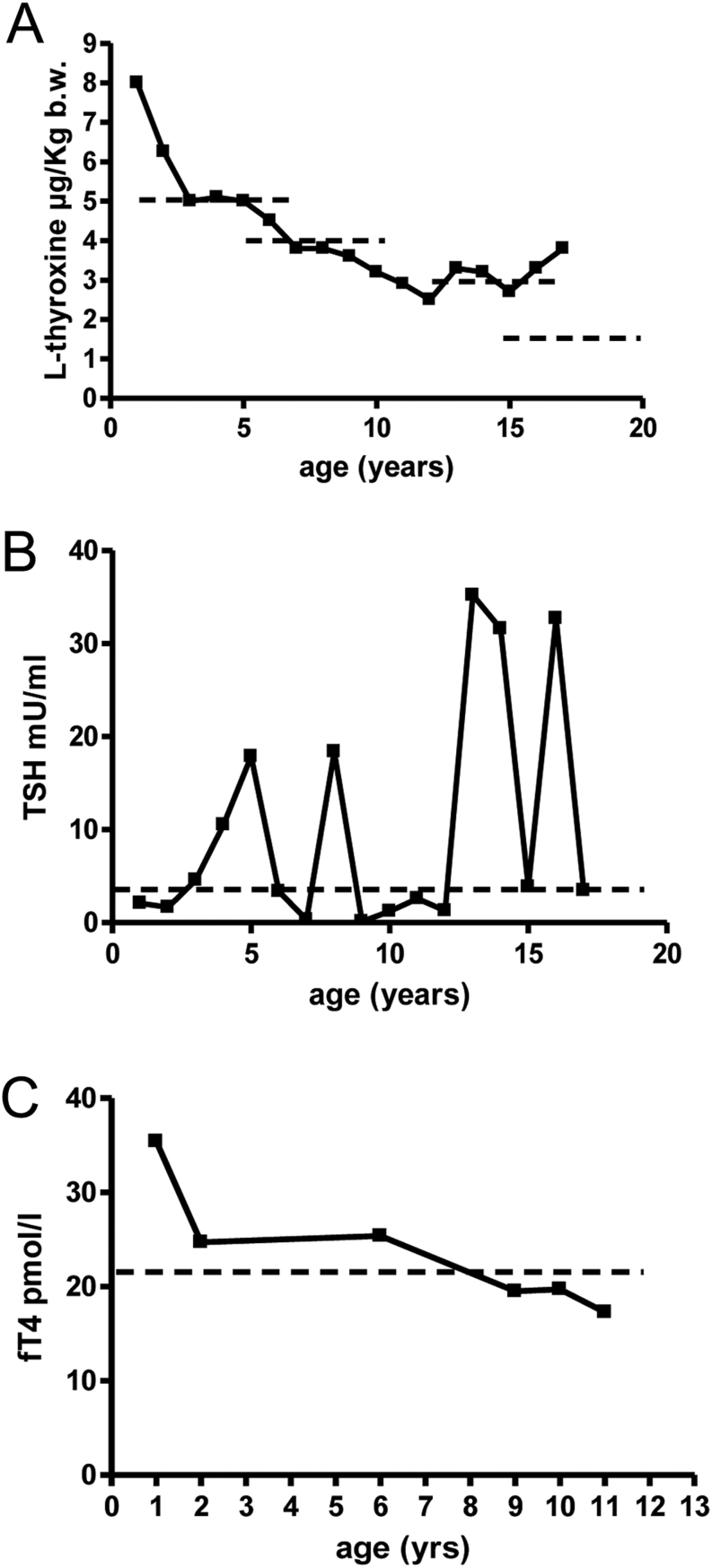
l-thyroxine requirement (A), TSH serum level (B) and free thyroxine trend over the years in patients 1. Dotted lines represent mean l-thyroxine requirement for age (A), upper normal limit for TSH (B) and the upper normal limit for fT4 (C).

**Figure 2:**
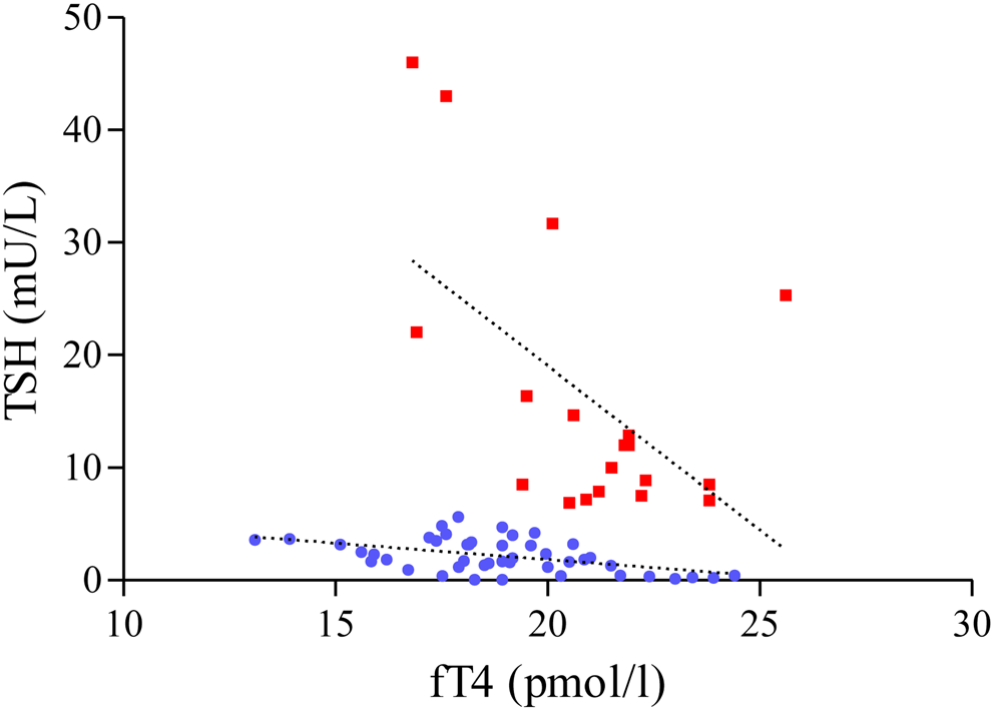
Scatter plot showing the linear regression (dotted lines) of TSH and fT4 values measured in 9 patients included in the study (red squares), compared with those of a control group of 15 congenital hypothyroid patients (blue circles). For each patients, a minimum of two determinations measured at different time during their follow-up are reported (RTH *n* = 19; controls *n* = 45). As shown in the figure, we did not found overlap between data from RTH and those of the controls.

A female born at term after an uneventful pregnancy, weight 3450 g and length 51 cm, was found to be hypothyroid at neonatal screening (TSH > 100 mU/L and fT4 = 3.7 pmol/L; normal values (nv) (12–22 pmol/L). No thyroid gland was visualized at scintigraphy. She was started on LT4 at the dose of 8 µg/kg body weight with a prompt normalization of fT4 and TSH. The dosage of LT4 was subsequently adjusted in order to maintain TSH and fT4 in the normal range, although high values of TSH were sporadically recorded and considered secondary to poor compliance. She grew along the 75th centile and had normal sexual development and menarche at 13.6 years. Subsequently, menses recurred regularly. Starting with 12 years, however, she required increasing doses of T4 to maintain TSH in the normal range (see Fig. 1B). The results of TSH determinations were confirmed by a different assay. Non-compliance or malabsorption was excluded. In addition, fT4 was always at or above the upper limit of the normal range (see Fig. 2). She never showed signs or symptoms of thyroid hormone excess such as tremors, tachycardia or sleep disorders. Acquired resistance to TH was thus suspected and a liothyronine (LT3) suppression test was performed (see Table 3). This test showed an incomplete suppression of TSH and a persistent response to i.v. TRH administration, confirming the suspected resistance to TH. At present time at the age of 18.6 years, her fT4 is 21.5 pmol/L (nv 12–22 pmol/L) and TSH is 3.45 mU/L (nv 0.2–4.5 mU/L) while receiving 225 µg daily of LT4, which correspond to a daily dosage of 3.6 µg/kg, the recommended doses being around 1.6 µg/kg body weight.

**Table 3:** Results of the LT3 suppression test in patient 1 (normal values: TSH (0.5–3.6), fT4 (**12–22**) (*9–20*), fT3 (3.5–7.7) (*4–8*)).

	Basal	3 days	6 days	9 days TRH test; basal/peak
LT4 (µg/day)	200			
LT3 (µg/day)		50	80	120
TSH (mU/L)	16.4	2.4	0.4	0.1/1.1
fT4 (pmol/L)	**19.5/***19.0*	**16.6**/*16.1*	**12.2**/*10.5*	**9.4**/*8.9*
fT3 (pmol/L)	**3.8/***4.9*	**7.9/***8.6*	**9.8**/*10.9*	**10.6**/11.5

Free TH values measured by Cobas are reported in bold and those by Delfia in italics.

Genetic analysis showed a heterozygous point variant of uncertain significance (VUS) in *GLIS3* gene (NM_152629) (*p.S298Y* MAF < 0.01) which was inherited from her euthyroid mother and a heterozygous point mutation in the *TSHR* gene (NM_000369) (p.T607I in exon 10), previously described in patients with CH (24).

### Case 2

The female was born at term after an uneventful pregnancy, weight 3260 g and length 48 cm, and was found to be hypothyroid at neonatal screening (TSH = 618 mU/L, fT4 = 1.5 pmol/L (nv 10–25 pmol/L). Thyroid scan was not performed, but an ultrasound showed a normal thyroid gland *in* *situ*. She was started on LT4 at the daily dose of 8–9 µg/kg body weight with a prompt normalization of the fT4 and TSH. She grew regularly between the 50th and 75th centile. At the first year of life, elevated TSH serum levels were observed (8–9 mU/L) despite fT4 levels in the high normal range (23.8 pmol/L; nv 10–24 pmol/L) and the patient being treated with an appropriate daily dosage of LT4 for chronological age (5 µg/kg). Lack of compliance with the treatment and malabsorption was excluded. Treatment required frequent corrections, but the patients continued to grow regularly, symptomless, with a normal bone age and neurological development. At the age of 7 years, because of the persistent elevation of TSH serum level (7.1 mU/L upper limit <3.6 mU/L) despite borderline high fT4 levels (23.9 pmol/L), resistance to thyroid hormone levels was suspected and an LT3 suppression test was performed, which confirmed a partial pituitary resistance (see Table 4). Sanger analysis of *THRB* and *GLIS3* gene was performed in this patient and we did not find any variant.

**Table 4:** Results of the LT3 suppression test in patient 2. Normal values: TSH (0.5–3.6), fT4 (**12–22**) (*9–20*), fT3 (3.5–7.7) (*4–8*).

	Basal	3 days	6 days	9 days TRH test; basal/peak
LT4 (µg/day)	75	75	75	75
LT3 (µg/day)		20	40	60
TSH (mU/L)	7.1	1.7	0.3	0.1/2.5
fT4 (pmol/L)	**23.8**/*21.7*	**22.9/***23.0*	**23.2**/*22.1*	**21.3**/*21.6*
fT3 (pmol/L)	**6.0**/*6.8*	**8.0**/*8.3*	**10.0**/*9.7*	**12.6/***11.0*

Free TH values measured by Cobas are reported in bold and those by Delfia in italics.

### Case 3

The patient born at term after an uneventful pregnancy, weight 3700 g and length 52 cm, was found to be hypothyroid at neonatal screening (TSH = 71 mU/L, fT4 = 2.7 pmol/L (nv 10–24 pmol/L). Thyroid scan showed normal thyroid gland *in situ*. She was started on lt4 at the daily dose of 7 µg/kg body weight with a prompt normalization of fT4 and TSH. She grew regularly between the 50th and 75th centile. At the third year of life, elevated TSH serum levels were observed (7.9 mU/L) despite fT4 levels in the high normal range (21.2 pmol/L (nv 10–24pmol/L) and the patients being treated with an appropriate daily dosage of lt4 for chronological age (8 µg/kg). Lack of compliance with the treatment and malabsorption was excluded. Currently, she is 13.2 years old, treated with 1.3 µg/kg and showed TSH serum levels between 8 and 30 mUI/L with FT4 serum values between 16.9 and 19.4 pmol/L (nv 12–22pmol/L). She also suffers from a mild cognitive defect. Genetic analysis was performed with the evidence of a double heterozygote allelic variant: a missense variant, p.E1546G (c.A4637G), in exon 34 of the *DUOX2* gene (NM_014080) and another missense variant, p.L597S (c.T1790C), in exon 16 of the *SLC26A4* gene (NM_000441). Both these variants were inherited from the euthyroid father.

### Case 4

A male born at term after an uneventful pregnancy, weight 3910 g and length 53 cm, was found to be hypothyroid at neonatal screening (TSH = 361 mU/L, fT4 = 10.2 pmol/L; nv 10–25 pmol/L). Thyroid scan showed an ectopic gland at sublingual position. He was started on lt4 at the daily dose of 10 µg/kg body weight with a prompt normalization of fT4 and TSH. He grew regularly between the 75th and 97th centile. At the second year of life, elevated TSH serum levels were observed (11–13 mU/L) despite fT4 levels in the high normal range (21.9 pmol/L; nv 12–22 pmol/L) and the patients being treated with an appropriate daily dosage of lt4 for chronological age (8 µg/kg). antibodies. At the last monitoring at the age of 6.4 years, his TSH value was 25.3 mU/L (0.7–6 mU/L) and fT4 25.6 pmol/L (12–22 pmol/L). He does not show either neurological or cognitive deficits.

### Case 5

A female born at term after an uneventful pregnancy, weight 3450 g and length 51 cm, was found to be hypothyroid at neonatal screening (TSH > 100 mU/L, fT4 = 3 pmol/L; nv 10–25pmol/L). Thyroid scan showed a normally structured thyroid gland in the right position. She was started on lt4 at the daily dose of 10 µg/kg body weight with a prompt normalization of fT4 and TSH. She grew regularly along the 50° centile, but at 14 months of age, excessive TSH serum levels were observed (46 mU/L) despite a normal fT4 serum level (16.8 pmol/L; nv 12–22 pmol/L). During the follow-up, TSH remained elevated with peaks (3.6 years) of more than 100 mU/L, with a fT4 always in the normal range. At the last monitoring at the age of 7.1 years, while receiving 75 µg/daily, her TSH value was 50.6 mU/L (0.7–6 mU/L) and fT4 16.5 pmol/L (12–22 pmol/L). She grows regularly along the 50th centile and does not show either neurological or cognitive deficits. Sanger sequencing of the *TSHR* gene (NM_000369) showed an heterozygous VUS (p.I155L in the exon 5 of the gene, MAF < 0.01).

### Case 6

A female born at term after an uneventful pregnancy, weight 2900 g and length 48.5 cm, was found to be hypothyroid at neonatal screening (TSH = 270 mU/L, fT4 = 6.1 pmol/L (nv 12–21.8 pmol/L)). Thyroid scan showed an ectopic gland at sublingual position. She was started on lt4 at the daily dose of 15 µg/kg body weight with a prompt normalization of fT4 and TSH. She grew regularly along the 25th centile, but at 4 years of age, raised TSH was observed (43.56 mU/L) together with a normal fT4 serum level (17.6; nv 12–22 pmol/L). During the follow-up, TSH remained elevated (≥9 mU/L) with a fT4 always at or above the upper limit. At the last monitoring at the age of 7.1 years, while receiving 50 µg/daily, her TSH value was 26.8 mU/L (nv 0.7–6 mU/L) and fT4 18.4 pmol/L (nv 12–22 pmol/L). She grows regularly along the 25th centile and doesn’t show either neurological or cognitive deficits. Molecular analysis by NGS identified no relevant variant.

### Case 7

A female born at term after an uneventful pregnancy, weight 2550 g and length 49 cm, was found to be hypothyroid at birth (TSH = 11.9 mU/L, fT4 = 19.3 pg/mL; nv 11.5–21.8 pmol/L). Thyroid scan showed a normal gland. She was started on lt4 at the daily dose of 2.5 µg/kg body weight with a prompt normalization of TSH. She grew regularly along the 50th centile (see figure), but at 3.5 years of age, a raised TSH was observed (7.22 mU/L) together with a normal fT4 serum level (20.9 pg/mL; nv 12–22 pmol/L). During the follow-up, TSH remained elevated (≥8 mU/L) with a fT4 always at or above the upper limit. At the last monitoring at the age of 4.8 years, while receiving 25 µg/daily, her TSH value was 11.3 mU/L (nv 0.7–6 mU/L) and fT4 21.3 pmol/L (nv 12–22 pmol/L). Thyroid ultrasound at the last visit showed a thyroid of normal size with a normal echographic pattern. She grows regularly along the 50th centile and doesn’t show either neurological or cognitive deficits.

Genetic tests showed heterozygous variants in the exon 6 of *DUOX2* gene (NM_014080) (p.G200R) and in exon 16 of *TPO* gene (NM_000547) (p.P906L). The parents declined additional investigations; thus, the inheritance of these variants is unknown.

### Case 8

A male born at term after an uneventful pregnancy, weight 3100 g and length 51 cm, was found to be hypothyroid at neonatal screening (TSH = 557 mU/L, fT4 = 2.12 pmol/L; nv 10–25 pmol/L). Thyroid scan showed a hypoplastic gland in the normal position. He was started on lt4 at the daily dose of 15 µg/kg body weight with a prompt normalization of fT4 and TSH. He grew regularly between the 3rd and 10th centile. At the second year of life, elevated TSH serum levels were observed (10–14 mU/L) despite high fT4 levels (21.8 pmol/L; vn 12–22 pmol/L) and the patients being treated with an appropriate daily dosage of lt4 for chronological age (2.5 µg/kg). At the last monitoring at the age of 8.2 years, his TSH value was 46 mU/L (0.27–4.2 mU/L) and fT4 19.1 pmol/L (12–22 pmol/L). He does not show either neurological or cognitive deficits. Molecular analysis by NGS identified no relevant variant.

### Case 9

A female born at term after an uneventful pregnancy, weight 3100 g and length 51 cm, was found to be hypothyroid at neonatal screening (TSH = 384 mU/L, fT4 = 2.4 pmol/L). Both scintigraphy and ultrasonographic examination failed to show a thyroid gland (athyreosis). X-rays at birth showed the absence of the distal femoral and proximal tibial epiphyses.

Replacement therapy with lt4 was started at the age of 9 days, at the daily dose of 12 µg/kg body weight with a prompt normalization of fT4 and TSH. She grew regularly along the 10th–25th centile, but at 15 months of age, a raised TSH was observed (22 mU/L; nv 0.7–6 mU/L) together with a normal fT4 serum level (16.9 pmol/L; nv 9.7–24.5 pmol/L). After increasing thyroxine dosage from 25 to 32 µg/day (2.6–2.9 µg/kg/day), both TSH and fT4 still resulted above reference values (6.76 µU/mL and 24.8 pmol/L). In view of high fT4 values, it was not considered appropriate to further increase the therapy. Subsequently, on several occasions, high TSH levels and fT4 levels in the upper part of the reference range were simultaneously detected. At the last monitoring at the age of 8.8 years, while receiving 64 µg/daily (2.6 µg/kg/day), her TSH value was 15.1 mU/L (0.27–4.2 mU/L) and fT4 21.11 pmol/L (12–22 pmol/L). She grows regularly 25h–10th centile and does not show either neurological or cognitive deficits. Molecular analysis showed two heterozygous VUS in the exon 33 (p.K1474E) and 34 (p.R1510H) of *DUOX2* gene (NM_014080) and two heterozygous VUS in the exon 15 of *TG*gene (NM_003235) (p.E1136Q and p.R1136Q).

## Discussion

We describe in this paper nine unusual patients, diagnosed at birth with different forms of CH, who developed, after a period of normal sensitivity, a pituitary resistance to TH over the years.

The diagnosis of RTHβ was suspected in all patients because of the contemporary and persistent elevation of fT4 and TSH, confirmed by repeated determinations in different laboratories. Thanks to the fact that all patients were followed by the same doctors (GR, FR, AS) in a prolonged follow-up, and we are fairly confident about the progressive reduction of tissue sensitivity to TH.

From the clinical point of view, patient 3 was also affected by a mild cognitive impairment implying a possible refractoriness at the CNS. In patients 1 and 2, a T3 suppression test was performed, followed by TRH test; these results are consistent with a partial pituitary resistance to TH action (Tables 3 and 4).

To our knowledge, such clinical picture of acquired resistance to LT4 has not been previously reported. Although some patients with CH may initially show some degree of hypothalamic–pituitary resistance to LT4, this condition usually improves over the years, while in our patients, it worsened (1, 17).

However, it has been reported that in about 40% of the patients with CH younger than 12 months and in 10% of older children, TSH fails to normalize despite an adequate LT4 treatment (1, 2).

Adults with CH retain a partial resistance to TH as they need higher doses of LT4 to maintain euthyroidism compared to those with adulthood-acquired hypothyroidism. A relative lack of thyroid hormones during the fetal life in some of these patients could determine this different set point (3) of the hypothalamus–pituitary–thyroid axis.

This set point may possibly change over the years for a physiological adaptive mechanism, similarly to what was reported in some elderly euthyroid patients. In fact, some authors have reported that pituitary sensitivity to TH may increase with age and about 3% of elderly outpatients, display an inappropriately low fT4 associated with normal TSH concentrations, not attributable to illness or drugs (25).

As a matter of fact, we do not have a reliable explanation for the illustrated findings of patients with normal sensitivity to TH in the first years of life and developing tissue resistance thereafter. However, as clearly shown in Fig. 2, there was no superimposition between the TSH values for a given fT4 serum level of our patients with those of a control group of children similarly affected by congenital hypothyroidism, this fact underpinning the diagnosis of resistance to thyroid hormones. Analytical interferences with the immunoassays were excluded since the results were confirmed by different laboratories and inadequate compliance with treatment or malabsorption has been excluded since the treatment has been always administered by the parents or under parental supervision and intestinal diseases were absent. Theoretically a change from a solid to liquid preparation might explain some differences in TSH serum levels; however, this does not apply to our patients who were all on tablet lt4.

Maternal factors influencing TH responsiveness seem a remote possibility. Indeed, the mothers of our patient were all euthyroid, they were not taking TH or other drugs that might have affected the thyroid function of their neonates. Moreover, RETH was not apparent at birth but developed later.

One possible mechanism can be related to the possible exposure to thyroid disruptors (e.g. phthalates) that during pregnancy, it has been associated with maternal FT4 levels, neonatal TSH (26, 27, 28) as well as thyroid function in adults (29). As an example, bisphenol A can act as a TH antagonist for TR binding, particularly potent on the beta isoform and can reduce hepatic DIO1 activity and compete with TH for the binding site on transport proteins (26, 27, 28, 29). However, the implications of such associations on public health have not been clearly established (29). Nevertheless, in the absence of stored samples to measure thyroid disruptors levels, we cannot support this hypothesis.

Another potential mechanism could potentially be an increased expression of the pituitary deiodinase type 3 (DIO3) gene, which encodes a membrane selenoenzyme that inactivates TH (9). But, the normal fT4/fT3 ratio (Table 2) would also exclude a possible alteration in the metabolism of TH.

In our opinion, the acquired tissue refractoriness to TH action is unlikely to be genetically determined as the decreased sensitivity to TH was observed in hypothyroid patients with different causes, either *in situ* or ectopic thyroid gland that would exclude a common genetic origin underpinning the findings. Accordingly, *THRB* was sequenced in all these patients and we did not find any variant in the entire coding region of the receptor. Moreover, none of the patients had variations in *SECISBP2*, *DIO1*or* DIO2* genes, including common SNPs (e.g. *DIO2* p.A92T) associated with impaired T4 to T3 conversion (30, 31). Moreover, in three patients, we did not find any genetic variants to explain the origin of CH. In four patients (#1, #3, #7, #9), who had athyreosis and an eutopic thyroid gland, we found double heterozygous missense variants in the *TSHR* and *GLIS3* (patient 1), *DUOX2* and *SLC26A4* (patient 3), in *DUOX2* and *TPO*genes (patient 7), and DUOX2 and TG (patient 9), configuring an oligogenic origin of the CH, as previously described by our group (21). However, none of these genetic variations is supposed to play a role in TH action.

Recently, Paone *et at.* described in a small group of CH patients the effectiveness of combined therapy of LT4/LT3 in restoring the biochemical control of circulating TSH, although it was unclear if this treatment also improved the neurodevelopmental outcomes of the patients (32). The results of the suppression test performed in two patients support the use of T4/T3 combined treatment as an alternative to LT4 alone in these patients.

One weakness of this study, due to its retrospective nature, relies on assays that were modified over the study period (more than 15 years) by the manufacturer and variable interventions performed in the patients at different ages, which might have biased the results. However, the longitudinal observations performed within the same subjects can, in our opinion, support the existence of acquired tissue refractoriness to TH action in these CH patients. This appears as an original observation, which deserves further research in order to identify the underpinning cause and that should be taken into consideration when clinical and biochemical discrepancies arise during the follow-up of CH patients.

## Declaration of interest

The authors declare that there is no conflict of interest that could be perceived as prejudicing the impartiality of the research reported.

## Funding

This work was funded by the Italian Ministry of Health.
